# A booster dose of Delta × Omicron hybrid mRNA vaccine produced broadly neutralizing antibody against Omicron and other SARS-CoV-2 variants

**DOI:** 10.1186/s12929-022-00830-1

**Published:** 2022-07-07

**Authors:** I-Jung Lee, Cheng-Pu Sun, Ping-Yi Wu, Yu-Hua Lan, I-Hsuan Wang, Wen-Chun Liu, Joyce Pei-Yi Yuan, Yu-Wei Chang, Sheng-Che Tseng, Szu-I Tsung, Yu-Chi Chou, Monika Kumari, Yin-Shiou Lin, Hui-Feng Chen, Tsung-Yen Chen, Chih-Chao Lin, Chi-Wen Chiu, Chung-Hsuan Hsieh, Cheng-Ying Chuang, Chao-Min Cheng, Hsiu-Ting Lin, Wan-Yu Chen, Fu-Fei Hsu, Ming-Hsiang Hong, Chun-Che Liao, Chih-Shin Chang, Jian-Jong Liang, Hsiu-Hua Ma, Ming-Tsai Chiang, Hsin-Ni Liao, Hui-Ying Ko, Liang-Yu Chen, Yi-An Ko, Pei-Yu Yu, Tzu-Jing Yang, Po-Cheng Chiang, Shang-Te Hsu, Yi-Ling Lin, Chong-Chou Lee, Han-Chung Wu, Mi-Hua Tao

**Affiliations:** 1 Institute of Biomedical Sciences, Academia Sinica, Taipei, Taiwan; 2 Graduate Institute of Microbiology, College of Medicine, National Taiwan University, Taipei, Taiwan; 3 Biomedical Translation Research Center, Academia Sinica, Taipei, Taiwan; 4 Institute of Cellular and Organismic Biology, Academia Sinica, Taipei, Taiwan; 5 Department of Clinical Laboratory Science and Medical Biotechnology, National Taiwan University, Taipei, Taiwan; 6 Genomics Research Center, Academia Sinica, Taipei, Taiwan; 7 Institute of Biological Chemistry, Academia Sinica, Taipei, Taiwan

**Keywords:** Omicron vaccine, mRNA vaccine, SARS-CoV-2, COVID-19, Variants of concern, Hybrid vaccine, Booster dose, Next generation vaccine, Cross-protectivity

## Abstract

**Background:**

With the continuous emergence of new SARS-CoV-2 variants that feature increased transmission and immune escape, there is an urgent demand for a better vaccine design that will provide broader neutralizing efficacy.

**Methods:**

We report an mRNA-based vaccine using an engineered “hybrid” receptor binding domain (RBD) that contains all 16 point-mutations shown in the currently prevailing Omicron and Delta variants.

**Results:**

A booster dose of hybrid vaccine in mice previously immunized with wild-type RBD vaccine induced high titers of broadly neutralizing antibodies against all tested SARS-CoV-2 variants of concern (VOCs). In naïve mice, hybrid vaccine generated strong Omicron-specific neutralizing antibodies as well as low but significant titers against other VOCs. Hybrid vaccine also elicited CD8+/IFN-γ+ T cell responses against a conserved T cell epitope present in wild type and all VOCs.

**Conclusions:**

These results demonstrate that inclusion of different antigenic mutations from various SARS-CoV-2 variants is a feasible approach to develop cross-protective vaccines.

**Supplementary Information:**

The online version contains supplementary material available at 10.1186/s12929-022-00830-1.

## Background

Since the COVID-19 pandemic occurred in late 2019, vaccines have been regarded as a major pharmaceutical intervention to combat the disease. Currently, global research and clinical efforts have pushed several COVID-19 vaccines approved for clinical use [1]. However, the pandemic still continues due to the constant emergence of new SARS-CoV-2 variants of concern (VOCs) [2]. Among the earlier identified VOCs, B.1.351 (Beta) exhibited the greatest immune escape against convalescent sera obtained from COVID-19 patients or vaccinated individuals [3]. The B.1.617.2 (Delta) variant that emerged in early December, 2020 quickly outpaced all other circulating isolates and significantly reduced vaccine efficacy [4]. Importantly, mutations in Delta strain enhances transmissibility among individuals and leads to more severe outcomes [5]. In late November 2021, the B.1.1.529 (Omicron) variant appeared and rapidly spread globally. This variant contains novel genomic sequence changes different from any of the previously defined ancestral or VOC isolates of SARS-CoV-2, including 37 mutations in the spike protein, 15 of which are located in the Receptor Binding Domain (RBD) [6]. Recent studies have shown that the increased number and complexity of spike mutations in the Omicron strain leads to its escape from therapeutic monoclonal antibodies [7–11]. Furthermore, constellation mutations render Omicron more antigenically distant from ancestral viruses or other VOCs, leading to reduced antibody neutralizing activity elicited by vaccination or natural infection [6, 8, 10–16]. Although the Omicron variant induces milder symptoms than Delta [17, 18], the higher transmission rate has inevitably led to an explosive increase in the case number and posed a big threat to the society. Therefore, it is pressing to develop new generation of COVID-19 vaccines that can effectively control VOCs pandemic.

In this study, we aim to develop vaccines targeting two currently major prevalent VOCs, Omicron and Delta, and a Hybrid RBD vaccine, which contained all 16 point-mutations of Omicron and Delta in a single construct to evaluate the effectiveness of vaccine predicting the potentially emerged variant that may evolve from the recombination event of these two predominant variants. We also tested the concept of Bivalent vaccines containing both Delta and Omicron RBD since multivalent vaccines containing various SARS-CoV-2 VOC antigens are recommended by the WHO Technical Advisory Group on COVID-19 Vaccine Components (TAG-CO-VAC) as a feasible approach to effectively control the spread of SARS-CoV-2 variants. We parallelly compared vaccine-elicited binding and neutralizing antibody titers and the T cell immunity against wild-type, Beta, Delta, and Omicron variants in mice which received a two-dose primary vaccination series or a third-dose booster further.

## Results

### Immunogenicity and protectivity of WT RBD mRNA vaccine

**Fig. 1:**
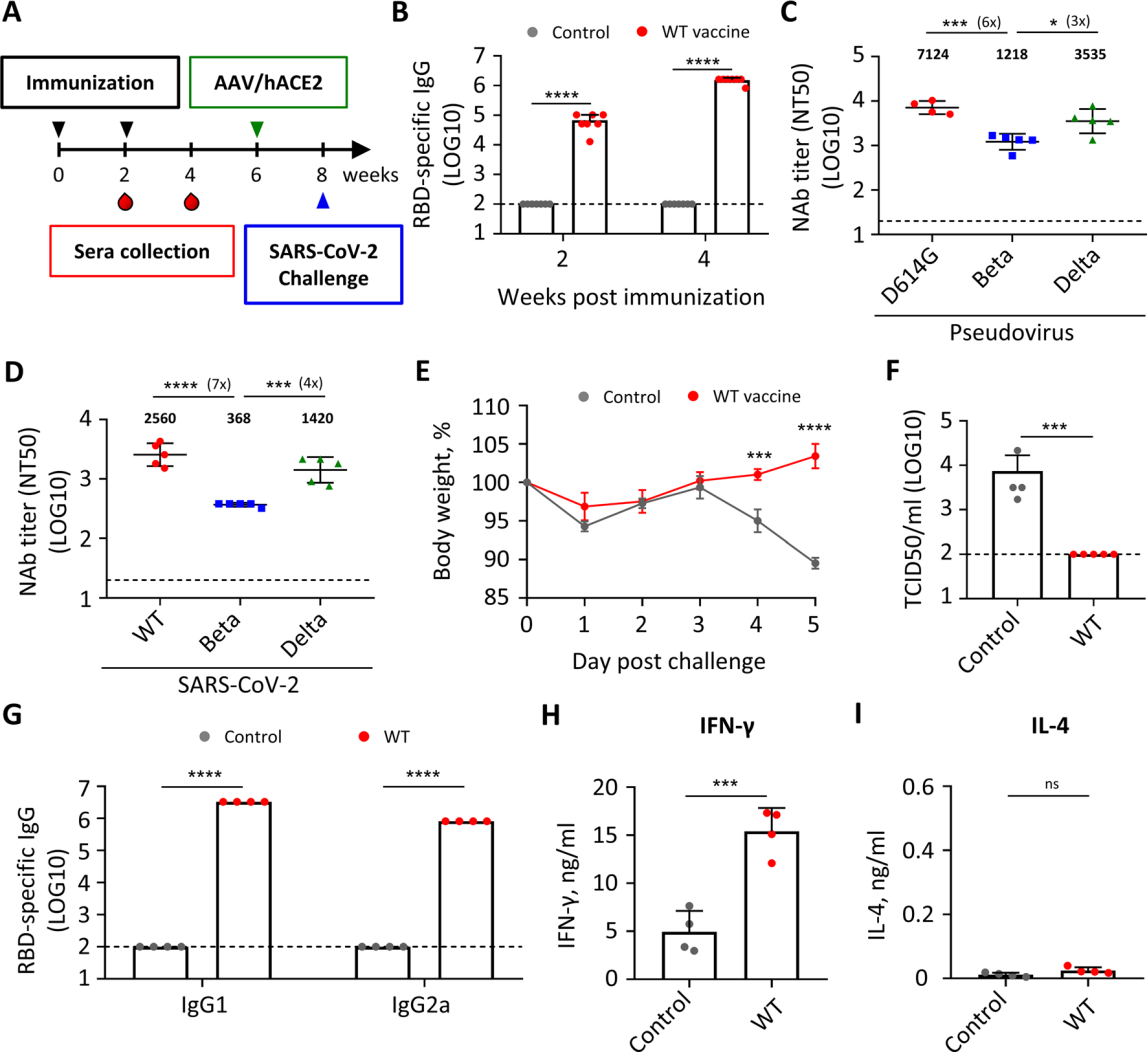
Wild-type RBD mRNA vaccine induces protective and Th1-biased immune responses against SARS-CoV-2. **A** Immunization, blood draw, AAV/hACE2 transduction, and wild-type SARS-CoV-2 (Wuhan strain) challenge schedule. **B** Serum IgG binding to recombinant SARS-CoV-2 RBD measured by ELISA. **C** Serum neutralizing activity against SARS-CoV-2 D614G, Beta, and Delta pseudovirus measured by pseudovirus neutralization assay. Plotted values represent geometric mean of 50% neutralizing titers (NT_50_). **D** Serum neutralizing activity against SARS-CoV-2 wild-type, Beta, and Delta virus measured by live virus micro-neutralization assay. Plotted values represent geometric mean of 50% neutralizing titers (NT_50_). **E** Body weight change of SARS-CoV-2 challenged mice. **F** Infectious viral load in lung of the challenged mice measured by Median Tissue Culture Infectious Dose (TCID_50_) assay 5 days post challenge. **G** Serum IgG1 or IgG2a binding to recombinant SARS-CoV-2 wild-type RBD measured by ELISA. **H**, **I** IFN-γ (**H**) and IL-4 (**I**) secretion of RBD-stimulated splenocytes of control and vaccinated mice measured by Multiplex assay. Statistical comparisons between control and vaccinated mice were determined by unpaired T test. Statistical comparisons across groups were determined by one-way ANOVA with Tukey’s multiple comparisons test. *p < 0.05, **p < 0.01, ***p < 0.001, ****p < 0.0001. ns, non-significant. Dotted line indicates the limit of detection. See also Additional file 1: Fig. S1

First, to examine the immunogenicity and protective efficacy of the RBD mRNA vaccine, we immunized naïve BALB/c mice twice over 2 weeks by intramuscular injection with the wild-type (WT, Wuhan strain) RBD vaccine and saline as controls (Fig. 1A). High titers of RBD-specific IgG antibodies were generated (Fig. 1B). SARS-CoV-2 pseudovirus neutralization assay showed that sera of vaccinated mice also had high titers of neutralizing antibodies against D614G and the Delta variant, with ~ sixfold lower titer against Beta variant (Fig. 1C). Similar finding was obtained in neutralization assay using authentic SARS-CoV-2 (Fig. 1D). The vaccinated mice were rendered SARS-CoV-2-permissive by subjecting to adeno-associated virus-transduced expression of human angiotensin-converting enzyme 2 (AAV/hACE2) 4 weeks post second immunization (Fig. 1A) [19] and 2 weeks later challenged with wild-type SARS-CoV-2. The WT vaccine could efficiently protect mice from body weight loss (Fig. 1E) and inhibit viral replication in lung (Fig. 1F). Our results confirmed findings of a previous report that WT RBD mRNA vaccine induced high antigen-binding and neutralizing antibody titers and conferred protection against SARS-CoV-2 infection [20]. The WT mRNA vaccine elicited a Th1-biased immune response as indicated by a balanced level of RBD-specific IgG1 and IgG2a (Fig. 1G) and high level of IFN-γ and no IL-4 secretion from stimulated splenocytes (Fig. 1H, I). In contrast, mice immunized with an alum-adjuvanted RBD protein vaccine induced high titers of RBD-specific IgG1 and limited amount of IgG2a (Additional file 1: Fig. S1A) and produced only IL-4 and no IFN-γ from stimulated splenocytes (Additional file 1: Fig. S1B, C), suggesting that alum-adjuvanted RBD protein vaccine tended to elicit Th2-biased immune responses. Taken together, these data demonstrate that our RBD mRNA vaccine induced potent immunogenicity, protectivity against SARS-CoV-2, and a Th1-biased immune response.

### Design and encapsulation of mRNA encoding WT and variant RBD

**Fig. 2:**
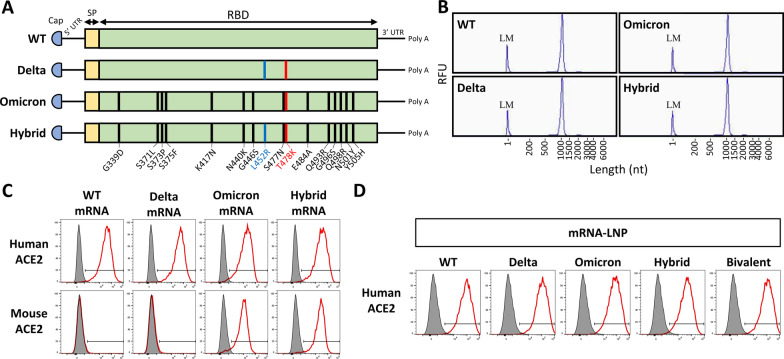
Characterization of WT and variant RBD mRNA and RBD mRNA-LNP vaccines. **A** Mutation sites of wild-type (WT), Delta, Omicron, and Hybrid RBD mRNA constructs. *UTR* untranslated region, *SP* signal peptide. **B** RNA identity and integrity of in vitro transcribed WT, Delta, Omicron, and Hybrid RBD mRNA measured by Fragment analysis. *LM* lower marker, *RLU* relative fluorescence units, *nt* nucleotide. **C**, **D** Binding capacity of WT and variant RBD expressed from mRNA (**C**) or mRNA-LNP (**D**) transfected cell supernatants to cells expressing human angiotensin-converting enzyme 2 (ACE2) or mouse ACE2 measured by flow cytometry. See also Additional file 1: Fig. S2

Four different mRNA vaccines were designed to encode the SARS-CoV-2 spike RBD region of the wild-type, Delta, Omicron, and Omicron with an additional L452R mutation found in Delta (named Hybrid) (Fig. 2A). In vitro transcription reaction was used to synthesize mRNA. Fragment analysis confirmed that the four synthesized RNA had expected length (around 1000 nt) and showed good integrity with 93% or 94% of intact RNA and only limited amounts of degraded transcripts (Fig. 2B and Additional file 1: Fig. S2A). The mRNA was then transfected to 293T cells to express various RBD and test for their binding capacity to cells that stably expressed either human or mouse angiotensin-converting enzyme 2 (ACE2). All WT, Delta, Omicron, and Hybrid mRNA expressed RBD efficiently bound human ACE2, while only Omicron and Hybrid RBD could bind mouse ACE2 (Fig. 2C). This result demonstrates that the RBD proteins translated from the synthesized mRNA were folded properly with correct conformation. This is consistent with previous reports that RBD of Omicron variant gained the ability to bind mouse ACE2 [6, 21]. The synthesized mRNAs were then packaged into lipid nanoparticles (LNPs) to form WT, Delta, Omicron, and Hybrid vaccines. The Bivalent vaccine was formulated with half dose of both Delta and Omicron mRNAs encapsulated into the same LNP. Dynamic light scattering (DLS) measurement showed that the average size of all mRNA-LNPs was around 90 nm with a narrow distribution (Additional file 1: Fig. S2B). All five RBD mRNA vaccines efficiently expressed RBD in transfected 293T cell supernatants (Fig. 2D).

### Immunogenicity of WT and variant RBD mRNA vaccines in naïve mice

**Fig. 3:**
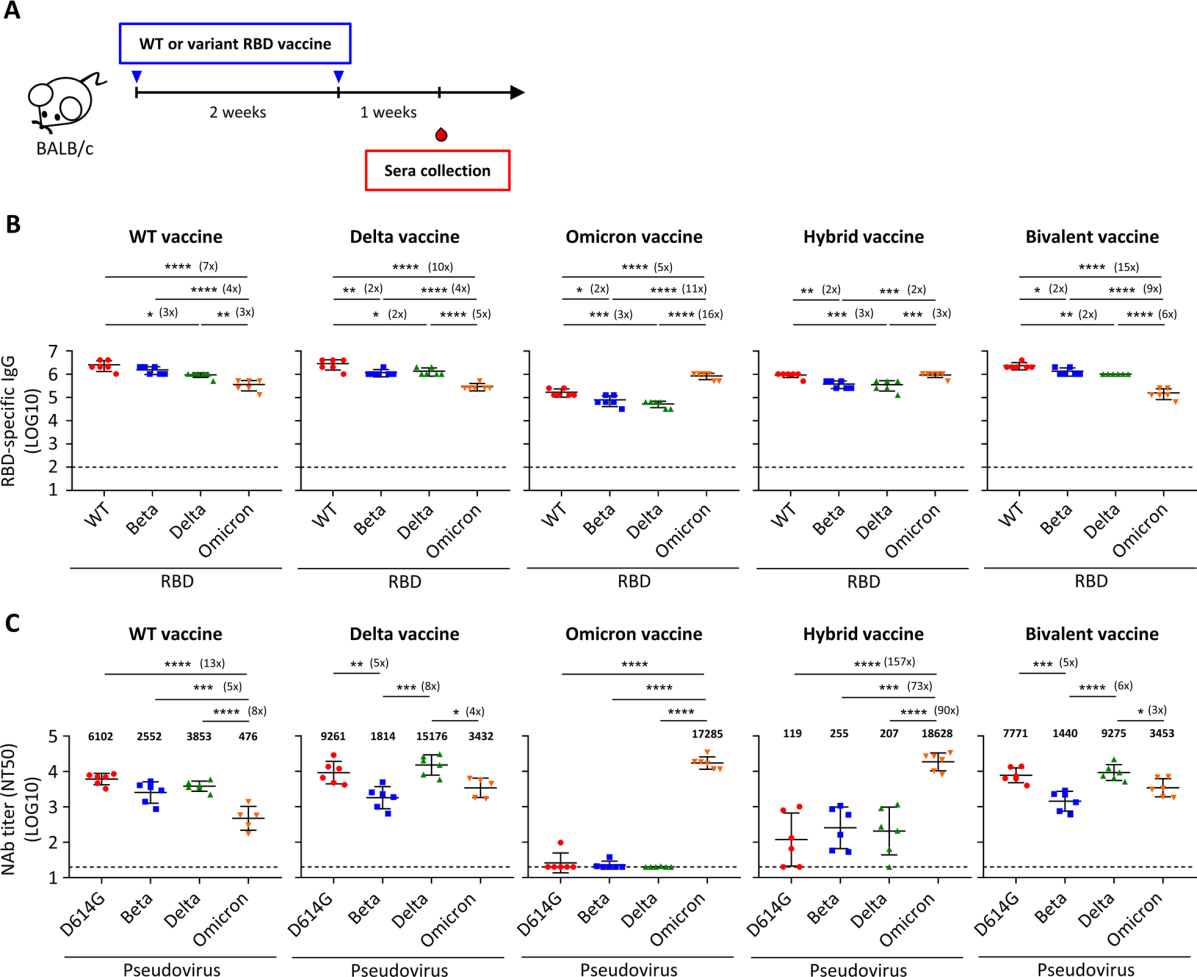
RBD-specific IgG binding and neutralizing antibodies in sera of naïve mice immunized by WT and variant RBD mRNA vaccines. **A** Immunization and blood draw schedule. **B** Serum IgG binding to recombinant SARS-CoV-2 RBD of WT, Beta, Delta, and Omicron strain measured by ELISA. Plotted values represent mean endpoint titers. Fold change between groups with statistically significance were shown after asterisks. **C** Serum neutralizing activity against SARS-CoV-2 D614G, Beta, Delta, and Omicron pseudovirus measured by pseudovirus neutralization assay. Plotted values represent geometric mean of 50% neutralizing titers (NT_50_). Fold change between groups with statistically significance were shown after asterisks. Statistical comparisons across groups were determined by one-way ANOVA with Tukey’s multiple comparisons test. *p < 0.05, **p < 0.01, ***p < 0.001, ****p < 0.0001. Dotted line indicates the limit of detection. See also Additional file 1: Fig. S3

The immunogenic effect of these WT and variant RBD mRNA vaccines was then examined in naive mice, which represented the unvaccinated population. Groups of naive BALB/c mice (n = 6) were immunized twice over 2 weeks and serum samples were collected 1 week post second immunization (Fig. 3A). Mice that immunized with the Hybrid vaccine generated high titers of RBD-specific antibody responses against WT as well as all tested variant RBD (Fig. 3B). The Omicron vaccine immunized mouse sera exhibited high titers of anti-Omicron RBD IgG, but 5–16-fold lower responses against WT, Beta, and Delta RBD. In contrast, the WT, Delta, and Bivalent vaccines elicited high IgG responses to WT, Beta, and Delta RBD, but 3–15-fold lower responses against the Omicron RBD. We also carried out an ELISA assay to measure binding antibody responses against WT and variant spike protein. Again, the Hybrid vaccine triggered the broadest spectrum of spike-binding antibodies among all the tested vaccines (Additional file 1: Fig. S3A). In sum, all of our WT and variant RBD mRNA vaccines were immunogenic and could stimulate RBD- and spike-specific antibody responses against WT and the variants.

Next, we assessed the ability of the various vaccines to generate neutralizing antibody responses against D614G and variant pseudoviruses. Sera collected from mice that were immunized with WT RBD mRNA vaccine showed high to moderate neutralizing capacity against the pseudovirus of D614G, Beta, and Delta variants with geometric mean NT_50_ values of 6102, 2552, and 3853 respectively (Fig. 3C and Additional file 1: Fig. S3B). However, the neutralization capacity against the Omicron variant was significantly lower with 5–13-fold decline (geometric mean NT_50_ of 476). In contrast, the Omicron RBD mRNA vaccine induced very high neutralizing antibody titers against the Omicron variant with a geometric mean NT50 of 17,285 but with almost undetectable neutralizing antibody titer against D614G and other tested variants. The Hybrid vaccine also stimulated extremely high titers of neutralizing antibodies against Omicron variant with a geometric mean NT50 of 18,628 and unexpectedly low but significant neutralizing antibody titers against D614G and other tested variants with geometric mean NT50 of 119, 255, and 207 against D614G, Beta, and Delta variant, respectively. The Delta and Bivalent vaccines elicited high titers of neutralizing antibodies against D614G and Delta variants and moderate responses to Beta and Omicron variants.

**Fig. 4:**
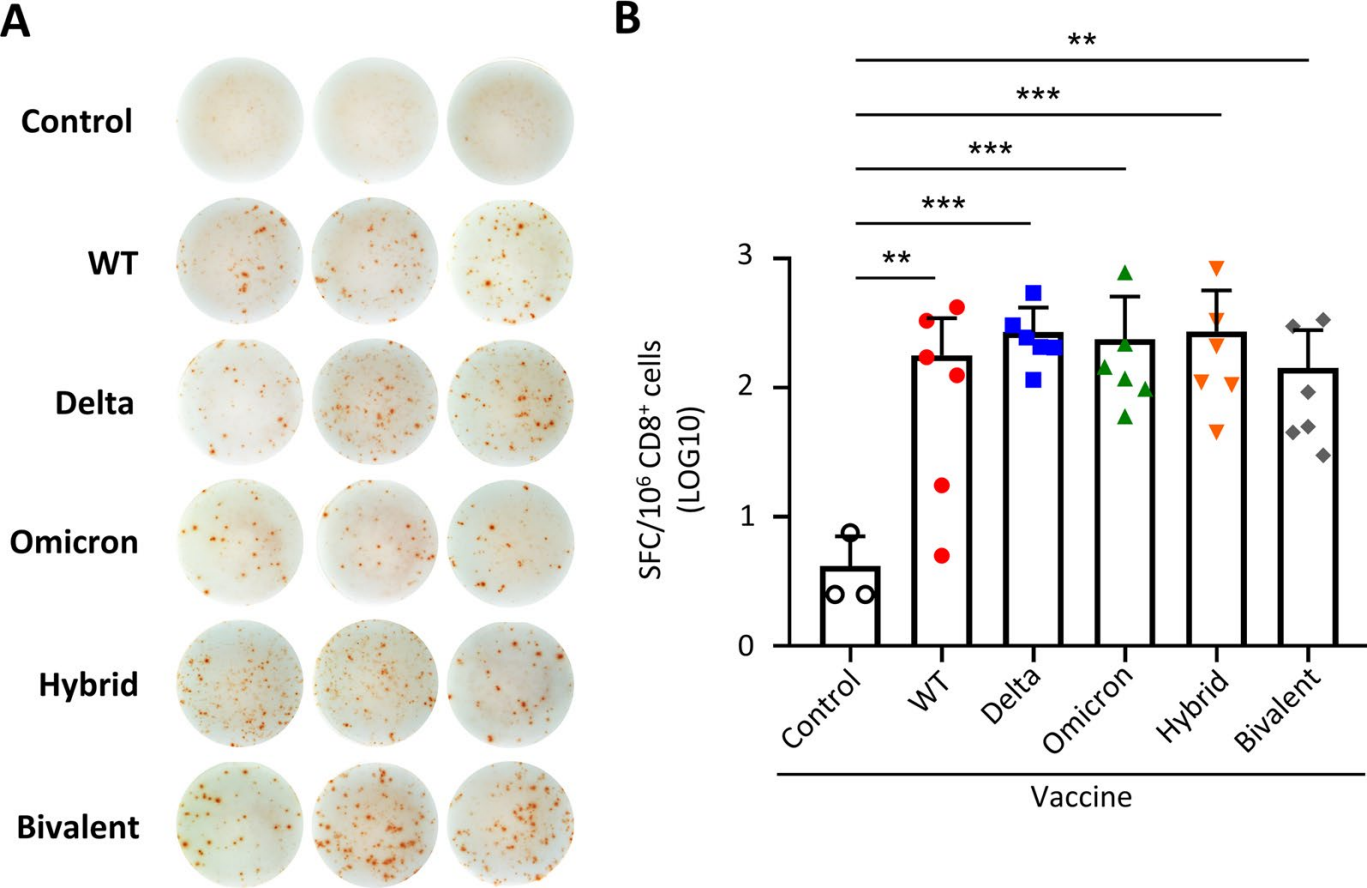
IFN-γ secretion of CD8^+^ cells in naïve mice immunized by WT and variant RBD mRNA vaccines. Mice were immunized with various RBD mRNA and the Bivalent vaccines as described in Fig. 3. **A**, **B** IFN-γ secretion capacity of CD8^+^ cells against SARS-CoV-2 (CoV-2) spike RBD peptide measured by ELISpot assay. Representative spot images were shown in **A** and summary spot counts shown in **B**. SFC, Spot forming cells. Statistical comparisons across groups were determined by one-way ANOVA with Tukey’s multiple comparisons test. *p < 0.05, **p < 0.01, ***p < 0.001, ****p < 0.0001. *ns* non-significant. See also Additional file 1: Fig. S4

Previous studies demonstrated that T cell responses to SARS-CoV-2 spike cross recognized Omicron variant [22–24]. We thus evaluated whether vaccination with different variant RBD mRNA vaccines could stimulate T cell responses with a conserved T cell epitope in the RBD protein [25]. Eighteen days post second immunization, CD8^+^ T cells were enriched from splenocytes of vaccinated mice (Additional file 1: Fig. S4) and stimulated with SARS-CoV-2 spike RBD peptide S526–533 [25]. ELISpot assay was conducted to evaluate the ability of RBD-specific T cells to secrete IFN-γ. The data showed that WT and all the variant RBD mRNA vaccines were able to stimulate RBD-specific CD8^+^ IFN-γ^+^ T cell responses recognizing a conserved T cell epitope (Fig. 4A, B).

### Immunogenicity of WT and variant RBD mRNA vaccines in vaccinated mice

**Fig. 5:**
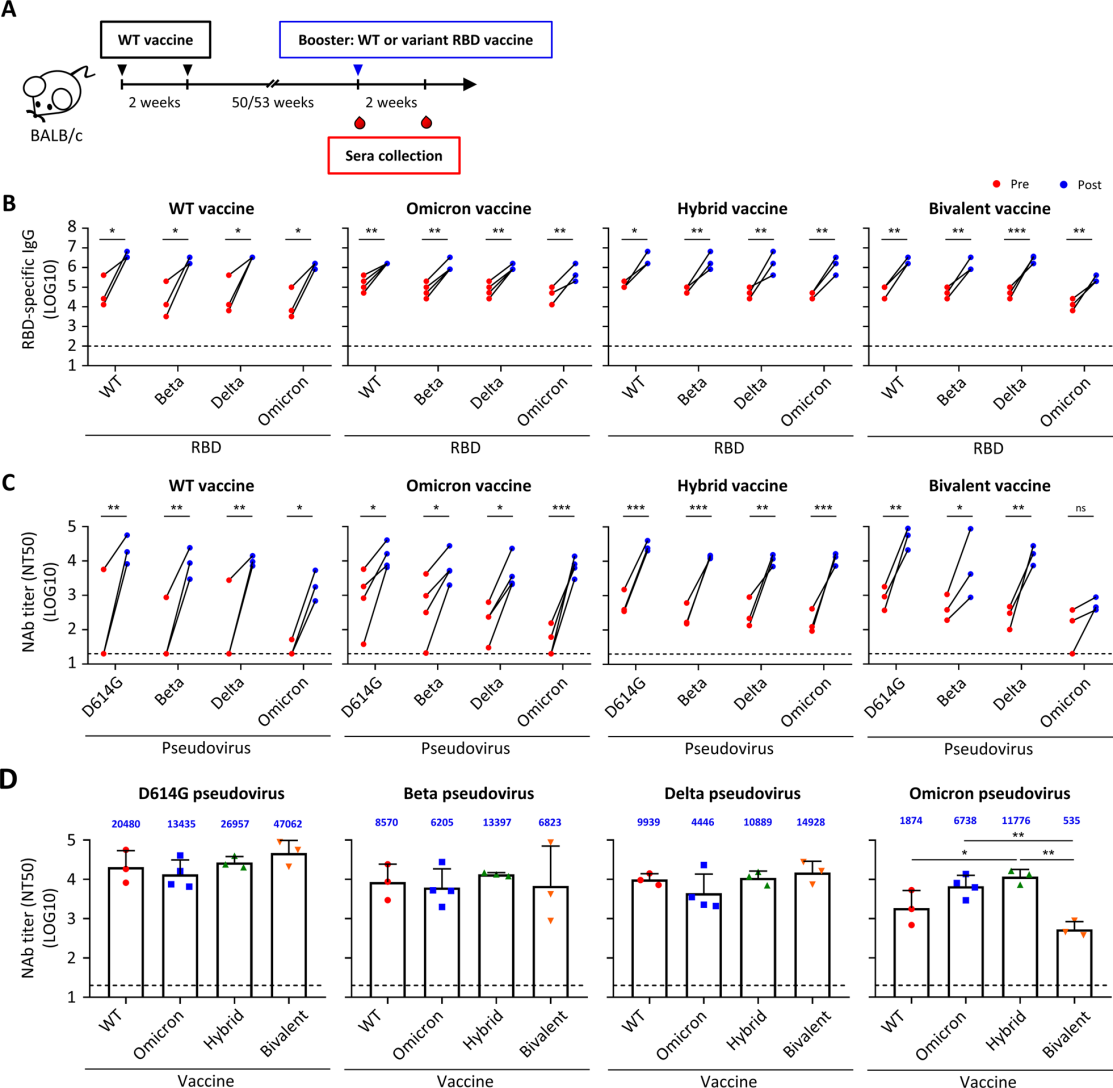
RBD-specific IgG binding and neutralizing antibodies in sera of long-term WT vaccinated mice boosted by WT and variant RBD mRNA vaccines. **A** Immunization and blood draw schedule. **B** Pre- and post-booster serum IgG binding to recombinant SARS-CoV-2 RBD of WT, Beta, Delta, and Omicron strain measured by ELISA. **C** Pre- and post-booster serum neutralizing activity against SARS-CoV-2 D614G, Beta, Delta, and Omicron pseudovirus measured by pseudovirus neutralization assay. **D** Post-booster serum neutralizing activity against SARS-CoV-2 D614G, Beta, Delta, and Omicron pseudovirus measured by pseudovirus neutralization assay. Plotted values represent geometric mean of 50% neutralizing titers (NT_50_). Statistical comparisons between pre- and post-booster were determined by repeated-measures two-way ANOVA with Sidak’s multiple comparisons test. Statistical comparisons across groups were determined by one-way ANOVA with Tukey’s multiple comparisons test. *p < 0.05, **p < 0.01, ***p < 0.001, ****p < 0.0001. Dotted line indicates the limit of detection. See also Additional file 1: Fig. S5

Currently, over 60 percentage of the world’s population has received at least one dose of COVID-19 vaccine. To determine the effect of booster dose on mice that had received prime doses for a long time period, we used cohorts of mice which had been immunized twice with 1 or 10 μg of WT RBD mRNA vaccine for 50 or 53 weeks (long-term WT vaccinated mice) and were randomly grouped and boosted with WT, Omicron, Hybrid or Bivalent RBD mRNA vaccines (Fig. 5A). Due to limited animal availability, three or four mice were used in each group and the Delta RBD mRNA vaccine was not included. Sera were collected 2 days before and 2 weeks after booster dose and subjected to RBD ELISA. Booster dose of WT and variant RBD mRNA vaccines could significantly boost RBD-specific IgG response against WT and all tested variant RBD in long-term WT vaccinated mice (Fig. 5B). Pseudovirus neutralization assay showed that neutralizing activities of pre-booster sera of mice that were immunized with WT vaccine for 1 year were at background or low levels against D614G and the variants (Fig. 5C and Additional file 1: Fig. S5A). This observation is consistent with the results that neutralizing antibodies waned in human sera over time [26]. Mice that boosted with WT vaccine showed high neutralizing responses against D614G, Beta, and Delta with a geometric mean NT_50_ value of 20,480, 8570, 9939 respectively, whilst response to Omicron variant was just moderate (NT_50_ value of 1874) (Fig. 5C and Additional file 1: Fig. S5B). Boosting by Hybrid vaccine induced high cross-reactive neutralizing antibodies against all D614G, Beta, Delta, and Omicron variants in long-term WT vaccinated mice, with the magnitude of neutralizing responses against Omicron the highest (NT_50_ value of 11,776) among all tested vaccines (Fig. 5D). Boosting with the Omicron vaccine also induced high neutralizing antibodies against D614G (NT_50_ value of 13,435), Omicron (NT_50_ value of 6738), and Beta (NT_50_ value of 6205) but moderate titers against Delta (NT_50_ value of 4446) variants. As to the Bivalent vaccine, neutralizing antibody titers were induced significantly against D614G, Beta, and Delta variants but not that apparently to the Omicron pseudovirus. Taken together, our results showed that boosting with WT and variant RBD mRNA vaccines in long-term WT vaccinated mice generates cross-reactive neutralizing antibody responses against all the tested variants, with Hybrid vaccine tends to induce the best antibody responses.

## Discussion

The emergence of highly contagious Omicron variant and its increasing incidences of breakthrough infection even after third-dose vaccination have raised great concerns about the protective efficacy of the currently available vaccines [27]. In addition, signal of a new variant, which designated as “Delta × Omicron Recombinant” identified in UK currently under monitoring has been announced on Feb 11, 2022 by the UK Health Security Agency, arousing high concerns in the world. The Omicron and Hybrid RBD generated from the mRNA vaccines showed high binding abilities to both human and mouse ACE2 (Fig. 2). This may be associated with the Q493R substitution present in both RBD as previously reported [6, 21]. Two other studies further showed that the Omicron variant could directly infect wild-type laboratory mice, although with limited weight loss and lower viral burden in the upper and lower respiratory tracts [28, 29]. This kind of change in RBD, which seemed to alter the viral tropism, highlights the need of specific booster for certain variants of concern.

By using a vaccinated mouse model which provided an identical genetic background and immune profile, we assessed the neutralizing antibody response induced by various RBD mRNA-LNP vaccines. Our results showed that WT RBD vaccine induced high antigen-binding, and neutralizing antibodies against D614G pseudovirus, which had been shown by Zhang et al. group [20], as well as Th1-skewed immune responses, which significantly suppressed virus replication and conferred protection from SARS-CoV-2 (Wuhan strain) infection in AAV/hACE2-transduced mouse model. WT vaccine can still induce high neutralizing antibody against Beta and Delta variants (Figs. 1D and 3B), but only caused a marginal effect (7.8% of D614G) to the Omicron variant (Fig. 3B), which echoed the human sera data [6, 10, 12–14]. The loss of WT vaccine-induced antibody responses against Omicron variant may be due to the loss of epitopes critical for neutralizing antibody recognition, as previously identified as mutations of K417N, G446S, E484A, and Q493R on the spike [9]. The Omicron-specific mRNA can induce high levels of antibodies recognizing recombinant RBD (Fig. 3B) and spike proteins (Additional file 1: Fig. S3A) of all strains, however antibody neutralizing activity was only limited to Omicron itself but not the other variants (Fig. 3C). This result was later confirmed by other reports [30, 31]. Hybrid mRNA also elicited good neutralizing effect to Omicron, with marginal responses on WT and other variants as well (Fig. 3C). Delta mRNA also induced cross-strain immunity against Omicron, similar to a recent report that Delta virus infection induced a cross-variant neutralization of Omicron [32]. The Bivalent vaccine also elicited cross-strain immunity in naïve mice, suggesting that this might be a promising strategy to increase the breadth and potency against SARS-CoV-2 variants.

Because at least half of the world population have received two doses of a COVID-19 vaccine, we are also interested in learning the boosting effects of the variant(s)-specific mRNA-LNP vaccines on this majority population. Thus, we also evaluated the immunogenicity of the mice who received two doses of WT RBD and about 1 year later boosted with either WT- or other VOC-based second-generation COVID-19 vaccines. Our results showed that all animals who received two doses of WT vaccine retained durable antibody binding ability but waning neutralizing antibodies over a year as previous reports described [26]. Promisingly, all mice who receive either WT or different variant booster vaccines significantly enhanced binding and neutralizing antibodies. In contrast to the limited neutralizing effects against D614G, Beta and Delta variants elicited by Omicron and Hybrid vaccines in naïve mice (Fig. 3C), these two vaccines as the booster induced a broad spectrum of neutralizing antibody titers against all SARS-CoV-2 strains (Fig. 5C), with the Hybrid vaccine being slightly better than the Omicron vaccine. Boosted with the WT vaccine also significantly increased neutralizing antibodies against all tested variants, but seems less well against Omicron compared with those boosted with Omicron and Hybrid vaccines, which was similar to the serum data collected from individuals who received a third dose of BNT162b2 vaccine. The cross-reactive immunity mediated by heterologous boosting was also confirmed by recent studies using Omicron vaccine-boosted mouse or non-human primate models [31, 33], or mRNA-1273-Beta boosting in participants having received a standard two-dose regimen of the mRNA-1273 vaccine [34]. The mechanism associated with the stimulations of cross-reactive humoral immune response induced by the third (booster) dose of mRNA vaccine was not clear at this time but may be related to the persistent germinal center B cell responses [35], continued B cell maturation and memory B cell generation in SARS-CoV-2 patients [36, 37]. Lastly, it may be noted that the Bivalent vaccine only induced comparably moderate neutralizing antibodies response against Omicron variant, especially as a booster in long-term WT vaccinated mice (Fig. 5C). This might be due to the underdosage of immunogen. On one hand, the Bivalent vaccine-immunized, long-term WT vaccinated mice were only exposed to one dose of Omicron mRNA-containing vaccine. On the other hand, comparing with the Omicron vaccine that contained full dose of Omicron mRNA and boosted high titer of anti-Omicron neutralizing antibody in long-term WT vaccinated mice, the Bivalent vaccine contained only half dose of the Omicron mRNA and may thus induced mild neutralizing antibody response against Omicron variant. Taken together, the dosage of the Omicron-specific mRNA in Bivalent vaccine may be one of the critical points to induce sufficient Omicron-specific neutralizing antibodies. This further emphasizes the importance of the composition or the ratio of different variant-specific mRNAs in the multivalent vaccine against SARS-CoV-2, which should be further studied.

## Conclusions

Omicron-specific mRNA as a priming dose induced a potent neutralizing antibody response against Omicron but not other SARS-CoV-2 variants. The monovalent Delta vaccine or the Bivalent vaccine will be a better option for people who have not got vaccination, while the Hybrid vaccine stands out as the best choice as a booster since it elicited broadly reactive neutralizing antibodies against Omicron and other variants. Our data provide some insights for rational design and choice of next generation vaccines which will be beneficial to unvaccinated population or people having received a standard two-dose regimen of currently approved vaccines.

## Materials and methods

### Animals

BALB/c mice were purchased from the National Laboratory Animal Center (Taipei, Taiwan) and maintained in a specific pathogen-free environment in the animal facilities of the Institute of Biomedical Sciences, Academia Sinica. All experimental procedures were reviewed and approved by the Animal Care and Use Committee of Academia Sinica.

### Generation of modified mRNA

DNA templates, which incorporated 5′ untranslated regions (UTR) (GGGAAAUAAGAGAGAAAAGAAGAGUAAGAAGAAAUAUAAGAGCCACC), signal peptide sequences from Igκ (ATGGAGACAGACACACTCCTGCTATGGGTACTGCTGCTCTGGGTTCCAGGTTCCACCGGTGAC), codon optimized wild-type (Wuhan-Hu-1, GenBank YP_009724390.1), Delta, Omicron, and Omicron with additional L452R mutation (Hybrid) RBD sequence, 3′ UTR (UGAUAAUAGGCUGGAGCCUCGGUGGCCAUGCUUCUUGCCCCUUGGGCCUCCCCCCAGCCCCUCCUCCCCUUCCUGCACCCGUACCCCCGUGGUCUUUGAAUAAAGUCUGA), and a poly-A tail were constructed. Before subjected to in vitro transcription reaction to synthesize mRNA with T7 RNA polymerase (NEB, MA, USA), the DNA template was linearized with EcoRV (NEB, MA, USA). The in vitro transcription reaction included CleanCap®Reagent AG (3′ OMe) (Trilink, CA, USA) for co-transcriptional capping of mRNA and complete replacement of uridine by *N*1-methyl-pseudouridine (Trilink, CA, USA). The mRNA was purified by LiCl (Invitrogen, MA, USA) precipitation and dsRNA was depleted by cellulose (Sigma-Aldrich, MA, USA). Purified RNA was kept frozen at − 80 °C until further use.

### Fragment analysis

RNA integrity was analyzed by fragment analysis following manufacturer’s protocol (Agilent, CA, USA). Briefly, mRNA was diluted to 2 ng/μl and mixed with diluent marker. RNA samples and ladder were denatured at 70 °C for 2 min and kept on ice before use. The percentage of RNA integrity was quantified by smear analysis using ProSize Data Analysis Software (Agilent, CA, USA).

### Preparation of RBD mRNA-LNP

The RBD mRNA was added to an ethanol solution containing a lipid mixture of cationic lipid, DMG-PEG2000 (MedChemExpress, NJ, USA), 1,2-distearoyl-sn-glycero-3-phosphocholine (DSPC) (Avanti, NY, USA), and cholesterol (Sigma, MA, USA). The weight ratio of the mRNA and the lipid in the ethanol solution was 3:1. The mixtures were subjected to the NanoAssemblr IGNITE™ NxGen Cartridges (Precision NanoSystems, BC, Canada) to produce mRNA-LNP composition, followed by treatments of dialysis against Dulbecco’s phosphate buffered saline (DPBS) (Gibco, MA, USA). The size and zeta potential of the mRNA-LNP were measured by Zetasizer Nano ZS (Malvern Panalytical Ltd., Malvern, WR, UK).

### RBD expression and binding assay

The variant-specific RBD mRNA was transfected into 293T cells via lipofectamine (Invitrogen, MA, USA) and the variant-specific RBD mRNA-LNP was transfected by direct addition. Cell supernatants were collected 2 days post transfection. To test the ability of RBD binding to human ACE2 or mouse ACE2, 293T-hACE2 or 3T3-mACE2 cells were harvested and aliquoted into FACS tubes at 5 × 10^5^ cells/tube. The cells were washed with staining buffer (DPBS + 1% BCS) and then incubated in 100 μl of transfected cell supernatant at 4 °C for 1 h. After washing, the cells were incubated with anti-RBD polyclonal antibody at 4 °C for 30 min. The cells were then washed two times, followed by 30-min incubation with PE-goat-anti-mouse IgG (H+L) antibody (Jackson ImmunoResearch, PA, USA) at 4 °C. The cells were washed twice and resuspended in 300 μl of staining buffer containing 7-AAD (Biolegend, CA, USA) for flow cytometry analysis (Thermo Fisher Attune NxT—14 color analyzer, Thermo Fisher Attune NxT software v2.2, FlowJo 10.6.1).

### Immunization

For naïve and long-term WT vaccinated mice immunization, groups of BALB/c mice were respectively immunized intramuscularly with two doses of WT (10 μg per dose), Delta (10 μg per dose), Omicron (10 μg per dose), Hybrid (10 μg per dose), and Bivalent (5 μg of both Delta and Omicron RBD mRNA per dose) vaccine with an interval of 2 weeks. The serum samples were collected from the mice 1 or 2 weeks post last immunization. The long-term WT vaccinated BALB/c mice used in booster dose experiments were obtained from two independent cohorts, which had been immunized with either 1 μg or 10 μg of WT RBD mRNA vaccine twice over 2 weeks.

### SARS-CoV-2 RBD-specific total IgG and IgG subclass ELISA

96-well plate (Thermo Fisher Scientific, MA, USA) were coated with 5 µg/ml of WT, Beta, Delta, or Omicron RBD or spike protein at 4 °C overnight. Plates were then blocked with 3% skim-milk/PBS at room temperature for 2 h. Serum samples were serially diluted and added to the blocked plates before incubation at room temperature for an hour. Following incubation, bound antibodies were either detected with goat anti-mouse IgG Fc HRP-conjugated antibody (Chemicon, CA, USA) for total IgG assessment or biotin-rat-anti-mouse IgG1 (BD Biosciences, NJ, USA) and biotin-rat-anti-mouse IgG2a (BD Biosciences, NJ, USA), and then followed by HRP- streptavidin (R&D Systems, MN, USA) for IgG subclass assessment. Plates were developed by TMB substrate (BD Biosciences, NJ, USA) and the reactions were stopped by adding 2N H_2_SO_4_. The absorbance at 450 nm were measured with EMax Microplate reader (Molecular Devices, CA, USA). The endpoint dilution titer was determined when titer value of the last serum dilution was twofold above the blank value.

### SARS-CoV-2 pseudovirus neutralization assay

293T cells that stably expressed human ACE2 (293T-hACE2) and lentiviral-based pseudotyped SARS-CoV-2 viruses were provided by National RNAi Core Facility (Academia Sinica, Taiwan). One day before neutralization assay, 293T-hACE2 cells were seeded into 96-well black plate (Perkin Elmer, MA, USA) at a density of 1 × 10^4^ cells per well at 37 °C. Mouse sera were inactivated at 56 °C for 30 min and then serially diluted by four folds with culture medium before incubation with indicated SARS-CoV-2 pseudovirus for an hour. The mixtures were then added to pre-seeded 293T-hACE2 cells and incubated for 3 days. Luciferase activity was measured by Luciferase Assay kit (Promega, WI, USA). The 50% neutralization titer (NT_50_) was calculated by nonlinear regression using Prism software version 8.1.0 (GraphPad Software Inc.).

### SARS-CoV-2 live virus micro-neutralization assay

Wild-type (hCoV-19/Taiwan/4/2020), Beta variant (hCoV-19/Taiwan/1013/2021), and Delta variant (hCoV-19/Taiwan/1144/2021) of SARS-CoV-2 virus were used to conduct live virus micro-neutralization assay and the experiments were performed in an approved biosafety level 3 (BSL-3) facility. Mouse sera were inactivated at 56 °C for 30 min and serially diluted by two folds before incubated with 100 TCID_50_ of wild-type, Beta, or Delta SARS-CoV-2 variant for an hour. The mixtures were then added to pre-seeded Vero E6 cells for 4-day incubation. Cells were then fixed with 10% formaldehyde and stained with 0.5% crystal violate for 20 min. The plates were washed with distilled water and scored for infection. The 50% neutralizing titer was calculated by Reed and Muench Method.

### SARS-CoV-2 challenge

Mice were anesthetized and transduced with 3 × 10^11^ vg of AAV6/hACE2 intratracheally and 1 × 10^12^ vg AAV9/hACE2 intraperitoneally 2 weeks after immunization [19]. The transduced mice were then challenged with 2 × 10^5^ TCID_50_ of SARS-CoV-2 (wild-type, hCoV-19/Taiwan/4/2020) intranasally. Mouse body weight was monitored daily. Five days post challenge, mouse lung was harvested for infectious viral load analysis. All animal experiments with SARS-CoV-2 challenge were conducted under animal biosafety level 3 (ABSL3) facility in Genomics Research Center, Academia Sinica (Taipei, Taiwan).

### SARS-CoV-2 viral load in lung

Lung tissues were homogenized in culture medium and clarified by centrifugation. Viral titers were determined in Vero-E6 cells monolayer grown in 96-well plates and tenfold serially diluted suspension was added to each well in quadruplicate. The plates were incubated in a CO_2_ incubator at 37 °C for 4 days, after which the cytopathic effects (CPEs) were observed microscopically at 40-fold magnification. The virus titer of each specimen, expressed as the TCID_50_, was calculated by the Reed and Muench method.

### Multiplex cytokine assay

The isolated splenocytes were cultured in RPMI 1640 medium containing 10% FBS, 50 μM β-mercaptoethanol, and 20 U/ml IL-2, and were stimulated with RBD-His recombinant protein at 10 µg/ml for 72 h. Cell supernatants were collected and the level of mouse IL-4 and IFN-γ in the supernatants was measured by Multi-Plex Immunoassay (MPI) performed by Inflammation Core Facility (Institute of Biomedical Sciences, Academia Sinica, Taipei, Taiwan). Antibody conjugated magnetic beads were incubated with cytokine-containing samples, washed, and incubated with biotinylated antibody and Streptavidin–Phycoerythrin (PE) subsequently. Fluorescence levels of the beads were measured by Bio-Plex® 200 system (Bio-Rad, CA, USA) and concentration of the cytokines was calculated with the standard. All assays were protected from light and performed at room temperature.

### CD8+ cell enrichment

Magnetic CD8 microbeads (Miltenyi Biotec, CA, USA) were used to enrich CD8^+^ cells from splenocytes of the vaccinated mice following the manufacturer’s instructions. In brief, cells were pelleted and resuspended in MACs buffer (PBS with 0.5% BSA and 2 mM EDTA). One hundred microliter of magnetic CD8 microbeads were added and incubated with 1 × 10^8^ splenocytes for 30 min at 4 °C. The unbound antibodies were removed and the cells were passed through MS columns (Miltenyi Biotec, CA, USA) that placed on an OctoMACS magnet (Miltenyi Biotec, CA, USA). Enriched cells were collected.

### Flow cytometry analysis

Total splenocytes and enriched CD8^+^ cells were stained on ice with anti-CD4 (BD Biosciences, NJ, USA), anti-CD8 (BD Biosciences, NJ, USA) antibodies in staining buffer (PBS with 1% FBS). Dead cells were excluded through the use of 7-AAD viability dye (Biolegend, CA, USA). FACSCanto (BD Biosciences, NJ, USA) was used to perform sample acquisition and FlowJo software (Tree Star, Inc., OR, USA) was used to analyze data.

### ELISpot assay

Mouse IFN-γ ELISPOT kit (eBioscience, CA, USA) was used according to the manufacturer’s protocol. Briefly, MultiScreen-HA 96-well plate (Millipore, MA, USA) was coated with IFN-γ-capturing antibody and blocked with 5% FBS/DMEM. Enriched CD8^+^ cells were then added at a density of 2 × 10^5^ cells per well and stimulated with 10 μg/ml SARS-CoV-2 spike peptide (S526–633) or irrelevant hepatitis B surface peptide (S28–39) in the presence of irradiated bone marrow-derived dendritic cells for 16 h. IFN-γ secretion was determined by biotin-rat anti-mouse IFN-γ for 2 h at room temperature. Plates were then washed and incubated with Avidin-HRP for 45 min. Plates were developed by AEC Substrate Solution and stopped by washing with distilled water. Dried plates were analyzed with AID vSpot ELISpot reader (AID Autoimmun Diagnostika GmbH, Strasburg, Germany) and accompanied software.

### Statistical analysis

Results are presented as the mean ± standard deviation (SD). Differences between experimental groups of animals were analyzed by unpaired T test, one-way ANOVA with Tukey’s comparison, or repeated-measures two-way ANOVA with Sidak’s multiple comparisons test. *p* < 0.05 was considered as statistically significant.

## Supplementary Information

**Additional file 1: Figure S1.** Alum-adjuvanted RBD protein vaccine induces a Th2-biased immune response. **Figure S2.** RNA integrity and basic characteristics of WT and variant RBD mRNA-LNP. **Figure S3.** Spike-specific IgG and neutralization curves of naïve mice immunized by WT and variant RBD mRNA vaccines. **Figure S4.** Representative enrichment rate of CD8+ cells in splenocytes of naïve mice immunized by WT and variant RBD mRNA vaccines. **Figure S5.** Neutralization curves of long-term WT vaccinated mice boosted by WT and variant RBD mRNA vaccines.

## Data Availability

All data generated or analyzed during this study are included in this published article and the supplementary information files. All other relevant data are available from the corresponding author on reasonable request.

## Abbreviations

COVID-19: Coronavirus Disease 2019
VOCs: Variants of concern
RBD: Receptor binding domain
WT: Wild-type
AAV: Adeno-associated virus
hACE2: Human angiotensin-converting enzyme 2
LNP: Lipid nanoparticles
DLS: Dynamic light scattering
