# Supplementary material for: A booster dose of Delta × Omicron hybrid mRNA vaccine produced broadly neutralizing antibody against Omicron and other SARS-CoV-2 variants
Source: J Biomed Sci. 2022 Jul 7;29:49. doi: 10.1186/s12929-022-00830-1 (PMC9261010; doi:10.1186/s12929-022-00830-1)
Supplement: Supplementary file 1 — Additional file 1: Figure S1. Alum-adjuvanted RBD protein vaccine induces a Th2-biased immune response. Figure S2. RNA integrity and basic characteristics of WT and variant RBD mRNA-LNP. Figure S3. Spike-specific IgG and neutralization curves of naïve mice immunized by WT and variant RBD mRNA vaccines. Figure S4. Representative enrichment rate of CD8+ cells in splenocytes of naïve mice immunized by WT and variant RBD mRNA vaccines. Figure S5. Neutralization curves of long-term WT vaccinated mice boosted by WT and variant RBD mRNA vaccines. [file 12929_2022_830_MOESM1_ESM.pdf]

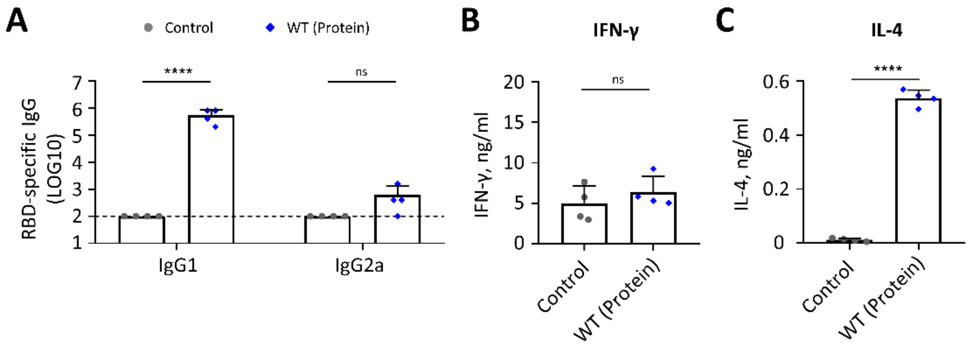

Fig. S1. Alum-adjuvanted RBD protein vaccine induces a Th2-biased immune response.

(**A**) Serum IgG1 or IgG2a binding to recombinant SARS-CoV-2 wild-type RBD measured by ELISA. (**B-C**) IFN-γ (B) and IL-4 (C) secretion of RBD-stimulated splenocytes of control and vaccinated mice measured by Multiplex assay. Statistical comparisons between control and vaccinated mice were determined by unpaired T test. * p<0.05, ** p<0.01, *** p<0.001, **** p<0.0001. ns, non-significant.

A

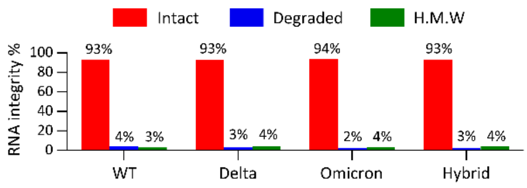

B

	Size (nm)	PdI	Zeta potential (mV)
**WT**	86.5±1.9	0.146±0.001	6.41
**Delta**	99.1±13.3	0.121±0.009	9.32
**Omicron**	91.1±3.7	0.136±0.010	6.48
**Hybrid**	92.1±4.7	0.143±0.017	6.93
**Bivalent**	91.2±10.2	0.147±0.035	8.96

Fig. S2. RNA integrity and basic characteristics of WT and variant RBD mRNA-LNP.

(**A**) RNA integrity of *in vitro* transcribed WT, Delta, Omicron, and Hybrid RBD mRNA measured by Fragment analysis. Plotted values represent the percentage of intact, degraded, and H.M.W. mRNA fragments of each preparation. H.M.W, High molecular weight. (**B**) Size, polydispersity index (pdI), and zeta potential of indicated RBD mRNA-LNP summary table. Data are presented as mean ± SD.

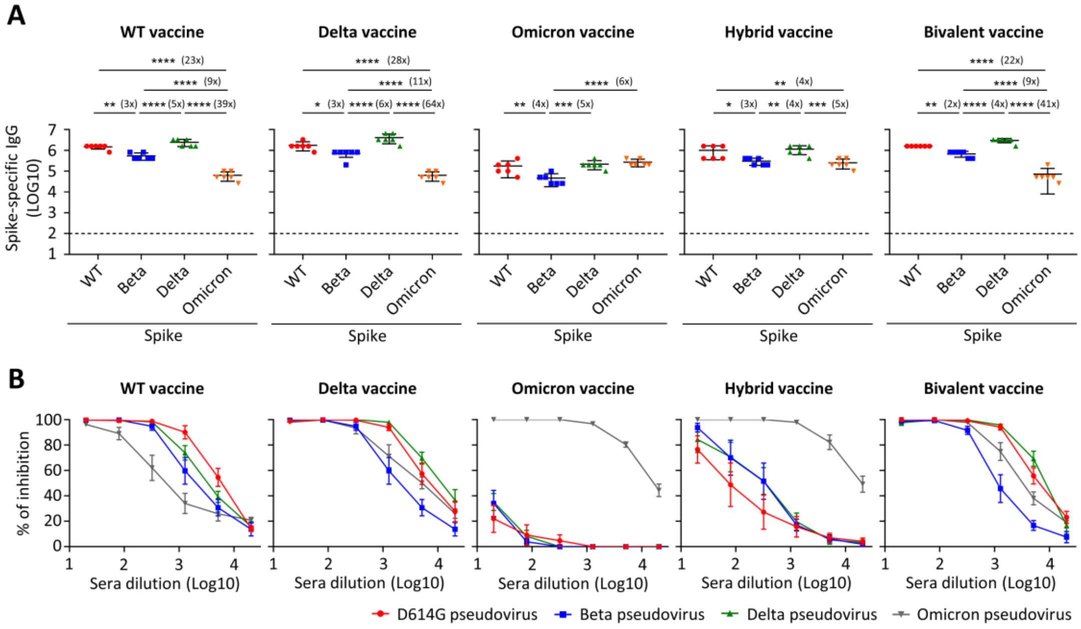

Fig. S3. Spike-specific IgG and neutralization curves of naïve mice immunized by WT and variant RBD mRNA vaccines.

Sera collection schedule was described in Fig. 3. (**A**) Serum IgG binding to recombinant SARS-CoV-2 spike of WT, Beta, Delta, and Omicron strain measured by ELISA. Plotted values represent mean endpoint titers. Fold change between groups with statistically significance were shown after asterisks. Statistical comparisons across groups were determined by one-way ANOVA with Tukey's multiple comparisons test. * $p<0.05$, ** $p<0.01$, *** $p<0.001$, **** $p<0.0001$. Dotted line indicates the limit of detection. (**B**) Serum neutralizing activity against SARS-CoV-2 D614G, Beta, Delta, and Omicron pseudovirus measured by pseudovirus neutralization assay.

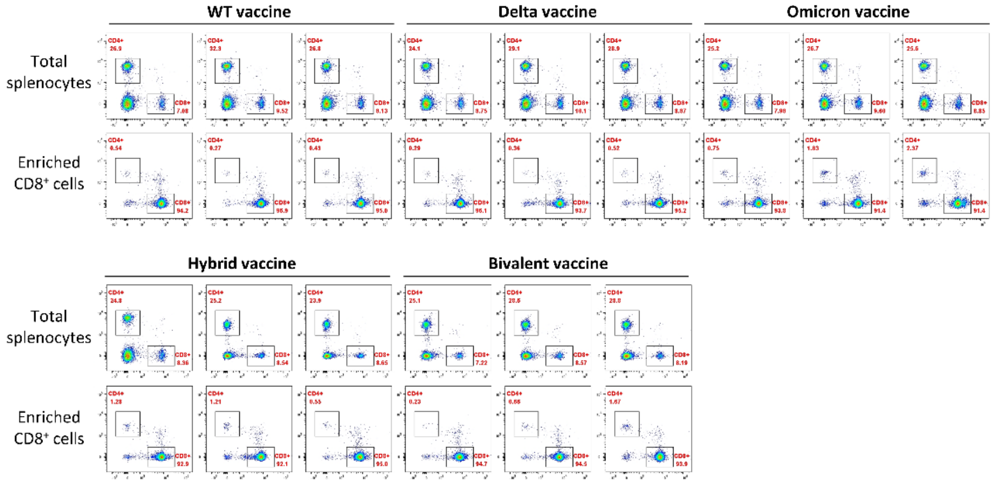

Fig. S4. Representative enrichment rate of CD8$^+$ cells in splenocytes of naïve mice immunized by WT and variant RBD mRNA vaccines.

Mice were immunized with various RBD mRNA and the Bivalent vaccines as described in Fig. 3. Splenocytes were collected from vaccinated mice 18 days post second vaccination. CD4$^+$ and CD8$^+$ cell percentages in total splenocytes and enriched CD8$^+$ cells in each group were measured by flow cytometry.

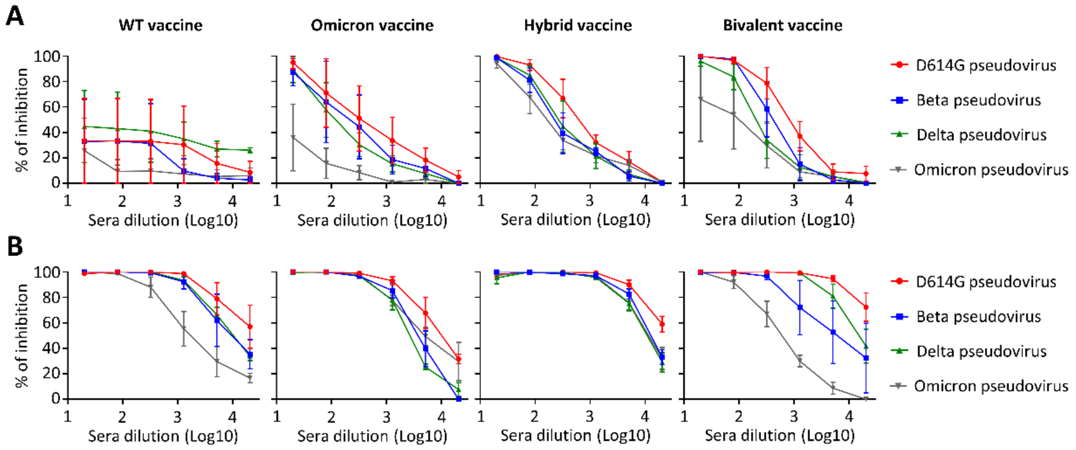

Fig. S5. Neutralization curves of long-term WT vaccinated mice boosted by WT and variant RBD mRNA vaccines.

Sera collection schedule was described in Fig. 5. (**A-B**) Serum neutralizing activity of pre- (A) and post-booster (B) vaccine mice against SARS-CoV-2 D614G, Beta, Delta, and Omicron pseudovirus measured by pseudovirus neutralization assay.
